# Ceftriaxone-Related Encephalopathy in a Patient With End-Stage Renal Disease and High Ceftriaxone Concentrations in Cerebrospinal Fluid and Plasma: A Case Report

**DOI:** 10.7759/cureus.46401

**Published:** 2023-10-03

**Authors:** Tomonori Takano, Mayumi Kaburagi, Satoru Morikubo, Daisuke Ichikawa, Kazuaki Matsumoto

**Affiliations:** 1 Department of Infectious Diseases, St. Marianna University School of Medicine, Kawasaki, JPN; 2 Department of Neurology, St. Marianna University School of Medicine, Kawasaki, JPN; 3 Division of Nephrology and Hypertension, Department of Internal Medicine, St. Marianna University School of Medicine, Kawasaki, JPN; 4 Division of Pharmacodynamics, Faculty of Pharmacy, Keio University, Minato, JPN

**Keywords:** nephrotic syndrome, cerebrospinal fluid concentration, hypoalbuminemia, end-stage kidney disease (eskd), ceftriaxone-associated encephalopathy, antibiotic-associated encephalopathy

## Abstract

Ceftriaxone (CTRX) does not require dose adjustment based on the renal function status and is used to treat infections. Recently, several studies reported the incidence of antibiotic-associated encephalopathy due to CTRX in patients with end-stage renal disease (ESRD). We experienced a case of CTRX-related encephalopathy in a patient on hemodialysis. When CTRX-related encephalopathy was discovered, the CTRX concentrations were measured in the blood and cerebrospinal fluid (CSF). The highest blood and CSF CTRX concentrations in this patient were 967 and 100.7 μg/mL, respectively, which were approximately 10 times higher than the CSF concentrations in a previously evaluated patient with CTRX encephalopathy. The concentration of CTRX may be increased in patients with ESRD. Hence, encephalopathy must be suspected in this patient group when CTRX is used.

## Introduction

Antibiotic-associated encephalopathy (AAE) is associated with the use of cephalosporins and metronidazole; among the cephalosporins, cefepime, and ceftazidime are known to cause encephalopathy [1]. Ceftriaxone (CTRX) is a third-generation cephalosporin that infrequently causes AAE, the number of patients who develop this condition is relatively small, and data on the CTRX concentrations measured in the blood and cerebrospinal fluid (CSF) are limited [2]. Herein, we experienced a case of CTRX-related encephalopathy (CRE) in a patient who underwent hemodialysis. We present the results of our analysis of the CTRX concentrations in the blood and CSF and discuss the pathophysiological mechanism of CRE in patients with end-stage renal disease (ESRD).

## Case presentation

**Figure 1:**
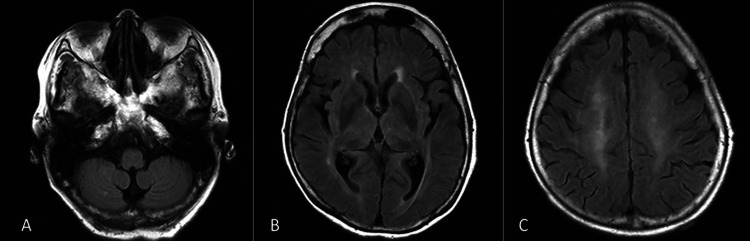
MRI of the brain. An axial fluid-attenuated inversion recovery-weighted (1.5 T; TR, 4,000 ms; TE, 106 ms) MRI of the brain shows the normal signal in (A) dentate nuclei, (B) thalamus, and (C) cerebral cortex. MRI, magnetic resonance imaging; TR, repetition time; TE, echo time

**Figure 2:**
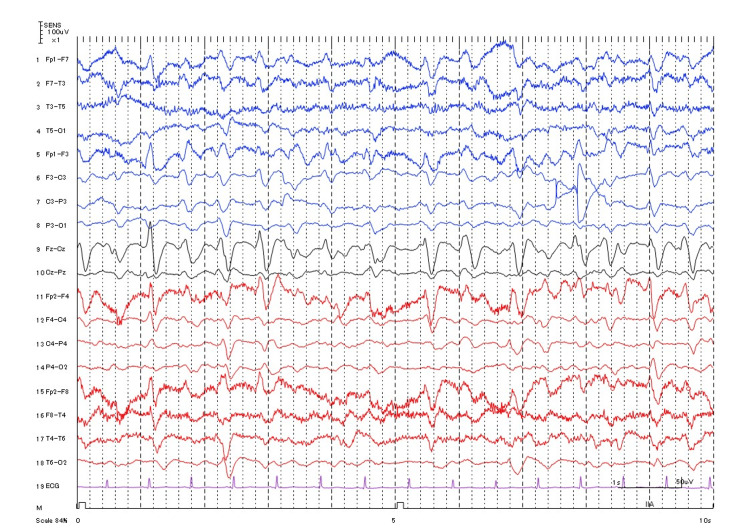
Electroencephalogram findings at the onset of impaired consciousness. ECG, electrocardiogram

We present the case of an 80-year-old woman. The patient was admitted to the hospital with complaints of weight gain and shortness of breath on exertion. Upon presentation to the hospital, the patient had a normal mental status, a weight of 77.6 kg (55.0 kg before the weight gain), and a height of 157 cm. The patient’s physical examination revealed a body temperature of 36.6 °C, a heart rate of 111 beats per minute, a blood pressure of 175/94 mmHg, a respiratory rate of 16 breaths per minute, and an oxygen saturation of 97% on room air. Except for the edema of the lower extremities, physical and neurological examinations yielded normal findings. Laboratory results showed a high albumin/creatinine ratio and low albumin (Table 1). Intermittent infusion hemodiafiltration was initiated on day 2 due to the decreased urine output associated with nephrotic syndrome. On day 6, the patient developed a fever (38.5 °C) and was treated with tazobactam/piperacillin (TAZ/PIPC). Blood cultures detected the presence of *Klebsiella oxytoca*, but the focus of the infection remained unknown; hence, the patient was treated with TAZ/PIPC until day 14. Based on the results of *K. oxytoca* drug susceptibility testing, TAZ/PIPC was discontinued on day 15 (eight days after the initiation of antimicrobial therapy), and intravenous CTRX was administered at a dose of 2 g per day. The Glasgow Coma Scale (GCS) score was E4V5M6 when CTRX was initiated, decreasing to E1V1M1 on day 16 (the day after CTRX was administered), indicating impaired level of consciousness. There were no abnormal neurological findings other than an impaired level of consciousness. In addition, her respiratory parameters (saturation and respiratory rate) and status were stable. Therefore, we did not use a ventilator. Brain magnetic resonance imaging (MRI) showed the absence of lesions (Figure 1); blood tests did not show electrolyte imbalance, hypoglycemia, or elevated ammonia levels; and spinal fluid tests showed normal findings. The electroencephalography (EEG) showed slow wave and triphasic waves with an anterior to posterior delay of 150 to 200 microV, synchronized from left to right (Figure 2). The EEG findings were typical for metabolic encephalopathy. Based on the clinical course, we suspected encephalopathy attributable to the newly initiated CTRX treatment. Consequently, CTRX therapy was discontinued on day 21 (seven days following the initiation of CTRX) as the patient exhibited symptoms of impaired consciousness.

**Table 1:** Laboratory results on admission.

Variables	Reference range	On admission
Urine albumin-to-creatinine ratio	<150 mg/g creatine	7,358 mg/g creatinine
White blood cell count	3.3 x 10^3^ to 8.6 x 10^3 ^μL^-1^	7.6 x 10^3 ^μL^-1^
Hemoglobin	11.6-14.8 g/dL	9.8 g/dL
Platelets	158 x 10^3 ^to 348 x 10^3 ^μL^-1^	351 x 10^3 ^μL^-1^
Total protein	6.6-8.1 g/dL	4.4 g/dL
Albumin	4.1-5.1 g/d L	1.2 g/dL
Aspartate aminotransferase	13-30 U/L	38 U/L
Alanine aminotransferase	7-23 U/L	22 U/L
Creatinine	0.46-0.79 mg/dL	2.86 mg/dL
Sodium	138-145 mEq/L	138 mEq/L
Potassium	3.6-4.8 mEq/L	5.3 mEq/L
Chloride	101-108 mEq/L	111 mEq/L
High-density lipoprotein cholesterol	48-103 mg/dL	49 mg/dL
Low-density lipoprotein cholesterol	65-163 mg/dL	327 mg/dL
Triglycerides	30-117 mg/dL	117 mg/dL
Glucose	73-109 mg/dL	92 mg/dL
C-reactive protein	0.00-0.14 mg/dL	0.70 mg/dL

**Figure 3:**
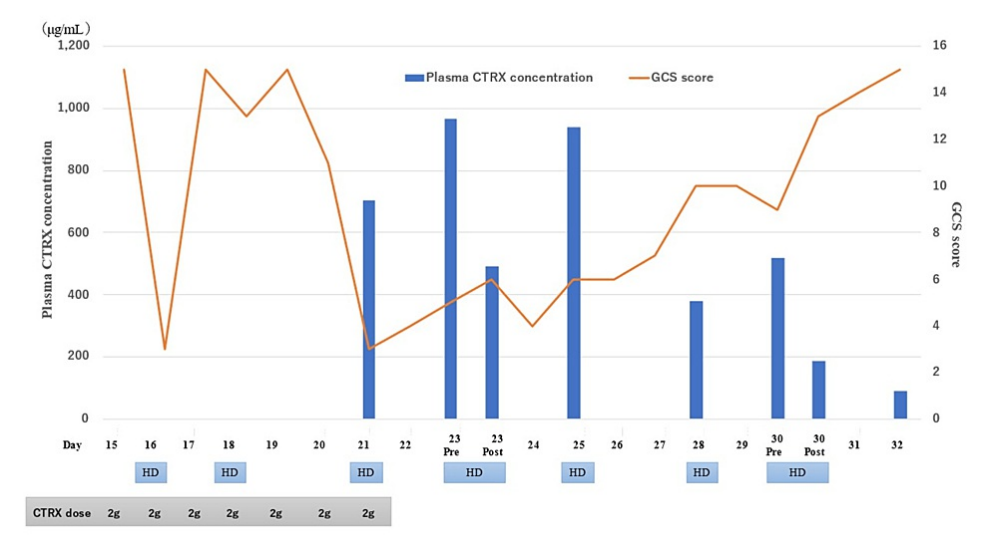
Clinical course and CTRX concentration. The plasma concentrations of CTRX were measured by high-performance liquid chromatography. The first day of hospital stay was defined as day 1. HD, hemodialysis; pre, pre-hemodialysis; post, post-hemodialysis; CTRX, ceftriaxone; GCS, Glasgow Coma Scale

When the CTRX concentrations in the blood and CSF were measured following the detection of impaired consciousness, the CSF CTRX concentration on day 22 was 100.7 μg/mL. On day 23 (the first day of CTRX discontinuation), the CTRX plasma concentration was 967 μg/mL, which was the highest value measured in the patient’s blood samples (Figure 3). Based on the EEG results and CTRX concentrations in the CSF and plasma, the patient was diagnosed with CTRX-induced AAE. On day 29 (the eighth day of CTRX discontinuation), the patient slowly regained the ability to speak and showed gradual improvements in her general condition without experiencing any sequelae.

## Discussion

AAE is divided into three categories according to its clinical phenotypes [1]. This case is classified as type 1 AAE. In type 1 AAE, seizures and loss of consciousness occur within a few days after the initiation of antimicrobial therapy. Type 1 AAE occurs with the administration of penicillin or cephalosporins and is characterized by abnormalities on EEG recordings but no findings on MRI [1,3]. The mechanism of type 1 AAE is being elucidated. Cephalosporins bind competitively to the gamma-aminobutyric acid class A receptor (GABAAR) 1 and block inhibitory neurotransmission at GABAAR, causing central excitotoxicity (disorientation and seizures) [1]. Type 2 AAE is characterized by psychiatric symptoms (delusions and hallucinations) within days of antimicrobial administration, often without abnormalities on EEG or MRI, and is caused by the use of fluoroquinolones or macrolide antimicrobials [1]. In type 3 AAE, cerebellar dysfunction (ataxia and movement disorder) occurs several weeks after starting metronidazole. Furthermore, MRI shows bilateral symmetric T2 hyperintense lesions in the cerebellar dentate nucleus, midbrain, dorsal pons, medulla, and splenium of the corpus callosum [1.3].

CTRX does not require drug dose adjustment based on the renal function status and is administered to elderly patients and patients with ESRD using the same dose [4]. Recently, CTRX encephalopathy has been reported in patients with ESRD [5,6-8]. Tan et al. demonstrated the effect of renal function (impaired renal function: estimated glomerular filtration rate with Cr and cystatin C [eGFRCr-Cys] = 10 mL/minute/1.73 m^2^; normal renal function: eGFRCr-Cys = 90 mL/minute/1.73 m^2^) and body weight on the peak concentration and area under the curve (AUC) of CTRX in the blood of elderly patients [9]. Compared with patients with normal renal function, those with impaired renal function have a threefold higher peak concentration and a fivefold higher AUC of CTRX. The peak concentration and AUC of CTRX are doubled in patients with a 35-kg body weight compared with that in patients with a 70-kg body weight. The peak concentration of CTRX may be sixfold higher, and the AUC may be ninefold higher in patients weighing 35 kg with impaired renal function compared with patients weighing 70 kg with normal renal function [9].

Previous studies indicated that the CSF concentrations of CTRX in CRE range from 6.6 to 10.2 μg/mL [4,8]. The CSF transfer of antimicrobials is enhanced at the onset of meningitis, and previous studies have shown a CSF CTRX concentration of 8 µg/mL in meningitis patients [10,11]. The CSF CTRX concentration in this patient was 100.7 μg/mL, approximately 10 times higher than that reported in previous studies. The plasma and CSF ceftriaxone concentrations were measured by high-performance liquid chromatography. Because the ceftriaxone concentration in the CSF was high, the same sample was measured six times (concentration range: 83.1-118.2 μg/mL), and the average value was calculated.

Unbound CTRX is free to move between the blood and CSF, but 90%-95% of the CTRX in the plasma is bound to protein [11]. Unbound CTRX is increased in patients with renal impairment [12]. In this case, the hypoalbuminemia was caused by nephrotic syndrome. Additionally, the patient had impaired renal function, which may have increased the unbound CTRX and CSF CTRX levels, leading to CRE. Ceftriaxone clearance is 10 times greater in patients who undergo dialysis than in those who do not undergo dialysis [13]. In this case, the concentration of ceftriaxone in the blood decreased by 50% after dialysis. However, the ceftriaxone concentration in the blood increased again two days after ceftriaxone was discontinued. In this case, the amount of interstitial fluid was increased due to nephrotic syndrome. In addition, the unbound ceftriaxone levels increased due to low albumin and ESRD. Unbound ceftriaxone can diffuse freely from the blood into the interstitial fluid; therefore, the blood concentration of ceftriaxone may have increased again due to the return of unbound ceftriaxone from the interstitial fluid into the blood [14].

## Conclusions

Older age, impaired renal function, and hypoalbuminemia are major risk factors for AAE. It should be noted that elderly patients often have impaired renal and hepatic function, reducing drug clearance. In renal impairment, in addition to decreased drug clearance, hypoalbuminemia occurs when there is a high loss of protein from the urine, as in nephrotic syndrome. Albumin-unbound antimicrobials are increased in hypoalbuminemia and can move freely between the blood and CSF. Therefore, the risk of AAE is increased in patients with renal dysfunction, such as nephrotic syndrome, and in those with hypoalbuminemia. In this case, it was thought that CTRX caused the AAE. CTRX has been used in the treatment of infectious diseases without the need for renal function adjustment of drug dosage. However, the blood levels of CTRX may be elevated in patients with ESRD, hypoalbuminemia, or low body weight, which may result in elevated CSF levels and possible development of encephalopathy. Therefore, patients with this condition must be closely monitored for the development of encephalopathy when CTRX is administered.

## References

[1] Bhattacharyya S, Darby RR, Raibagkar P, Gonzalez Castro LN, Berkowitz AL (2016). Antibiotic-associated encephalopathy. Neurology.

[2] Nishioka H, Cho Y, Irie K, Kanamori M (2022). Ceftriaxone-associated encephalopathy in a patient with high levels of ceftriaxone in blood and cerebrospinal fluid. Int J Infect Dis.

[3] Kim E, Na DG, Kim EY, Kim JH, Son KR, Chang KH (2007). MR imaging of metronidazole-induced encephalopathy: lesion distribution and diffusion-weighted imaging findings. AJNR Am J Neuroradiol.

[4] MUNAR MY, SINGH H (2007). Drug dosing adjustments in patients with chronic kidney disease. Am Fam Phys.

[5] Suzuki S, Naito S, Numasawa Y (2019). Encephalopathy induced by high plasma and cerebrospinal fluid ceftriaxone concentrations in a hemodialysis patient. Intern Med.

[6] Inoue Y, Doi Y, Arisato T, Sugioka S, Koga K, Nishioka K, Sugawara A (2017). Three cases of hemodialysis patients receiving high-dose ceftriaxone: serum concentrations and its neurotoxicity. Kidney Int Rep.

[7] Safadi S, Mao M, Dillon JJ (2014). Ceftriaxone-induced acute encephalopathy in a peritoneal dialysis patient. Case Rep Nephrol.

[8] Onogi C, Osada A, Imai K (2022). Two cases of ceftriaxone-induced encephalopathy treated by hemoperfusion in hemodialysis patients. Hemodial Int.

[9] Tan SJ, Cockcroft M, Page-Sharp M (2020). Population pharmacokinetic study of ceftriaxone in elderly patients, using cystatin C-based estimates of renal function to account for frailty. Antimicrob Agents Chemother.

[10] Goldwater PN (2005). Cefotaxime and ceftriaxone cerebrospinal fluid levels during treatment of bacterial meningitis in children. Int J Antimicrob Agents.

[11] Nau R, Sörgel F, Eiffert H (2010). Penetration of drugs through the blood-cerebrospinal fluid/blood-brain barrier for treatment of central nervous system infections. Clin Microbiol Rev.

[12] Joynt GM, Lipman J, Gomersall CD, Young RJ, Wong EL, Gin T (2001). The pharmacokinetics of once-daily dosing of ceftriaxone in critically ill patients. J Antimicrob Chemother.

[13] Tsai D, Zam BB, Tongs C (2023). Validating a novel three-times-weekly post-hemodialysis ceftriaxone regimen in infected Indigenous Australian patients-a population pharmacokinetic study. J Antimicrob Chemother.

[14] Levison ME, Levison JH (2009). Pharmacokinetics and pharmacodynamics of antibacterial agents. Infect Dis Clin North Am.

